# Impact of drinking Chinese green tea on postoperative short outcomes for gastric cancer: a randomized controlled trial

**DOI:** 10.1038/s41430-021-00868-8

**Published:** 2021-03-19

**Authors:** Dan Liu, Xinxin Jing, Shougen Cao, Xiaodong Liu, Xiaojie Tan, Haitao Jiang, Zhaojian Niu, Mengmeng Su, Jian Zhang, Xingqi Zhang, Gan Liu, Yanbing Zhou

**Affiliations:** 1 Department of Gastrointestinal Surgery of Affiliated Hospital of Qingdao University, Qingdao, China; 2 Department of Paediatrics of Affiliated Hospital of Qingdao University, Qingdao, China

**Keywords:** Randomized controlled trials, Gastric cancer, Gastric cancer

## Abstract

**Background:**

Early intake after surgery can decrease postoperative ileus. Several studies show coffee can stimulate bowel activity and be safe in patients after elective colectomy, mainly due to caffeine. It was postulated that drinking Chinese green tea as rich caffeine beverage after subtotal distal gastrectomy accelerates postoperative recovery in patients.

**Method:**

This was a single-centre parallel open-label randomized trial. Patients with gastric cancer undergoing robotic or laparoscopic subtotal gastrectomy were randomly allocated to receive drinking Chinese green tea (GT group) or potable water (PW group) after surgery. The primary endpoint was the time to gastrointestinal function recovery and tolerance of solid food, and the secondary endpoints included the incidence of postoperative complications, symptoms of postoperative adverse reaction, length of stay, pain as assessed by analgesic consumption and a visual analogue scale, and fatigue as assessed by a fatigue score model.

**Results:**

A total of 80 patients were recruited, 40 to each group. Patient characteristics were similar in both groups. The GT group showed significantly shorter time to gastrointestinal function recovery compared with PW group to first flatus (47.23 ± 13.46 vs. 76.96 ± 20.35, *P* < 0.001), first bowel motion (78.70 ± 25.77 vs. 125.76 ± 36.25, *P* < 0.001) and tolerance of solid food (62.20 ± 16.15 vs. 98.66 ± 20.15, *P* < 0.001).

**Conclusion:**

Drinking Chinese green tea after robotic or laparoscopic subtotal gastrectomy is safe and promotes postoperative recovery of gastrointestinal function, also was an add method with strengthening analgesia and anti-inflammatory effect in the presence of the Enhance Recovery After Surgery (ERAS) program. Registration number: ChiCTR1800018294 (http://www.chictr.org.cn).

## Introduction

Gastric cancer is the fifth most common cancer and the third leading cause of cancer-related deaths worldwide [1], and surgical resection is the only appropriate treatment to improve the survival rate of gastric cancer patients [2]. Howerer, gastric cancer surgery remains a high-risk procedure and the morbidity and mortality rates after radical gastrectomy have been reported as 12.5–18.3% and 0.5–1.2%, respectively [2, 3]. Postoperative ileus, the most common pathophysiological state after gastrectomy, increases length of hospital stay and hospital costs, which overload the economic burden of public health. How to promote the recovery of patients, reduce the incidence of postoperative complications and reverse the stress state of patients has become a hot spot in modern medical research.

Enhance Recovery After Surgery (ERAS) is a multidisciplinary protocol of care delivered to patients with the aim of maintaining normal physiology and thereby facilitating postoperative recovery [4, 5]. Based the original work of Kehlet and Wilmore [6], the ERAS protocol has been widely accepted and applied in the clinic. Our center have finished a trial to probe the application of ERAS in perioperative gastrectomy [7] and we found that early intake of liquid and food after surgery can facilitate postoperative restoration without increasing the incidence of fistulas, consistent with other studies [8–11]. However, it is controversial what kind of food or liquid should be the standard of early intake. Several studies show coffee can stimulate bowel activity in patients after elective colectomy, mainly due to caffeine [12–14], but there are few reports on the effects of beverages on accelerating recovery after gastrectomy.

Green tea (GT) is commonly consumed in China, Japan, and Eastern Asia. Rich in catechins, various amino acids, and caffeine, it possesses diverse pharmacological properties, which include anti-oxidative, anti-inflammatory, hypocholesterolemic, anti-arteriosclerotic, and anti-bacterial [15]. The daily consumption of GT is safe for the normal population [16, 17]. The gastrointestinal tract is most likely to be affected by tea drinking, since it has direct contact with the tea solution and its components, usually in high concentrations, irrespective of whether they are absorbed, retained or recirculated to the gut tissues. Therefore, we hypothesized that drinking Chinese GT could improve short outcomes in patients who underwent laparoscopic or robotic subtotal distal with gastrectomy.

## Methods

### Inclusion and exclusion criteria

From September 2017 to May 2018, all patients aged 18–75 years who suffered from gastric cancer underwent Robotic or laparoscopic subtotal distal gastrectomy (D2 Roux-en-Y) without preoperative neoadjuvant therapy at Department of Gastrointestinal Surgery of the Affiliated Hospital of Qingdao University.

The following criteria excluded the patients from trial participation.

1. Participation in a concurrent interventional trial
2. Complete pyloric obstruction
3. Known hypersensitivity or allergy to caffeine, coffee, or tea
4. American Society of Anesthesiologists (ASA) Physical Status Score of IV or V
5. Alcoholism or drug abuse
6. Previous extensive abdominal surgery (any open or laparoscopic abdominal surgery except laparoscopic appendectomy, cholecystectomy, or hernia repair)
7. Inflammatory bowel disease
8. Cardiac insufficiency more than III grade (NYHA)
9. Intake of opioid analgesics, or steroids >5 mg/day for ≥7 days before surgery
10. Long-term history of intake of glucocorticoid or gastrointestinal motility drugs
11. Intake of liver dysfunction medicine
12. Under anti-depressive medication
13. Primary diabetes and impaired glucose tolerance, hyperthyroidism or hypothyroidism
14. Severe obesity (BMI > 32) and severe malnutrition BMI < 15)
15. History of constipation
16. Pregnancy, lactation, or childbearing potential without using adequate contraception

### Surgery

Surgery was performed by the same surgical team. Now lots of evidences show that there is no significant difference about robotic vs. laparoscopic gastrectomy for gastric cancer in postoperative complications and short-and long-term outcome [18–21]. The surgery method about laparoscopic or robotic surgery was chose by patients and family with the intention-to-treat principle at the time of operative consent. All patients underwent standard D2 gastrectomy, with reconstruction of Roux-en-Y gastrojejunostomy, aiming to reduce anastomotic tension and prevent bile regurgitation. The operating procedure and lymph node dissection were planned on the basis of the Japanese gastric cancer treatment guidelines (ver. 4) [22]. Operative duration was time from skin preparation until dressing placement.

### Anesthesia

Anesthesia was standardized epidural anesthesia (Th7)–assisted general anesthesia, with no premedication, sufentanyl (Sufenta, IDT Biologika GmbH, Germany) for analgesia, Propofol (Diprivan, AstraZeneca, Britain) for induction, and Atracurium (Tracrium, The Wellcome Foundation Limited, Italy) or vecuronium bromide (Norcuron, Organon, Holland) as muscle relaxant. Epidural anesthesia medicine includes 70 ml 2% lidocaine (Xylociane, QiLu, Chian), 70 ml 1% ropivacaine (Naropin, AstraZeneca, Britain) and 50 μg sufentanyl with pump speed of 2 ml/h, which will be removed at 3 days after surgery.

### Perioperative care

All patients in the trial were placed on an established, standardized enhanced recovery after surgery program (see Table 1).

**Table 1:** The perioperative ERAS protocol.

Measure	Details
Preoperative	
Prehibilitation	Yes
Information and counseling about the surgery	Information about ERAS protocols and MDT
Bowel preparation	No
Preoperative fasting	Normal meal until 6 h before surgery
Carbohydrate loading	500 ml of 10% carbohydrate 2 h before surgery
Nasogastric tube	No nasogastric tube before surgery
Checklist and Timeout	Yes
Intraoperative	
Minimally invasive surgery (Lap./Rob.)	Yes
Incision size	Small incision in premise of fully exposed to the surgical field
Anesthesia	A combination of epidural analgesia (Th7-8) and general anesthesia
Intraoperative warming	Thermal insulation blanket during the surgery, Rinsing intraperitoneally with warm saline after surgery
Antibiotic use	Using once 30 min before surgery, the additional one if surgery lasts more than 3 h
Postoperatve	
Abdominal drainage	Removed 24 h after surgery
Incision suture	Absorbable suture without stitches
Analgesia postoperatively	Subcutaneous injection of long-acting local anesthetic drug in the incision, epidural analgesia postoperatively, i.v. nonsteroidal anti-inflammatory drugs (NSAIDs)
Urinary catheter	Removed 24 h after surgery
I.v. infusion of liquid	About 2000 ml per day
Mobilization	Encourage patients to mobilize in bed the day of surgery, mobilize out of bed the first day after surgery
DVT prevention programs	Antithrombotic stockings, Application of Antithrombotic pump during the intraoperative and postoperative, Prophylactic heparin, early ambulation
Diet reintroduction	Oral intake of a little clear water as soon as effects of anesthesia disappeared, following a stepwise plan from oral liquid food to normal diet, supplemented with parenteral nutritional

### Intervention

We chose Laoshan Mountain GT. The ratio of material to liquid was 1:200. Tea was brewed only once by 80-degree-Celsius water for 5 min, followed by filtering and cooling to a suitable temperature (room-temperature about 25–30 °C) for drinking. For the GT group (GT group), 500 ml (same as 2.5 g tea) was administered on the first day after operation, which increased from the second day to 1000 ml (same as 5 g tea), which was maintained until discharge. The potable water group (PW group) was provided the same volume of PW with room-temperature. During the trial additional fluids were allowed, but other beverages, including sodas, milk, yoghourt, coffee, and fruit juice, were not. Oral feeding after surgery followed ERAS program, from liquid food to solid food based on the tolerance of food with safety principle.

### Primary outcomes and secondary outcomes

The primary outcome was recovery of gastrointestinal function. This was measured by time to first flatus, time to first bowel motion and time to tolerance of solid food [23]. Time to toleration of solid food was defined as the time to solid food ingestion with no significant nausea or vomiting for 4 h. Secondary outcomes included the incidence of postoperative complications, symptoms of nausea, vomiting, diarrhea and bloating, postoperative hospital stay, postoperative pain as measured by analgesic consumption and a visual analog pain (VAS) scales, and postoperative fatigue as measured by a fatigue score model. Complications were graded according to the Clavien-Dindo classification [24].

### Date collection

Prospective demographic data collection included age, sex, comorbidities, ASA grade, previous surgery, surgical indication, NSR2002 (Nutrition risk screening, NSR), smoking history, comorbidities and body mass index. Perioperative data collected included surgery method, blood loss, intraoperative liquid volume, operative duration, total narcotic requirements, and intraoperative complications.

Data were collected by an independent investigator not involved with clinical management during the postoperative period on a daily basis. The primary outcomes of time to first flatus and time to first bowel motion, GT tolerance and the symptoms of nausea, vomiting, and bloating were recorded by the patient, which was collected daily by the investigator. VAS pain scores and fatigue scores were recorded daily at this appointment. Comparison of fatigue scores [25] and VAS scores was performed daily, whereas analgesic consumption was recorded as NSAID milligrams, with daily rates analyzed and compared. Fasting blood samples were collected before operation and 1 day, 3 days and 5 days after surgery to detect biochemical parameters, inflammatory factors which include leucocyte count, percentage of neutrophils, C-reactive protein (CRP), procalcitonin (PCT), interleukin-6 (IL-6) and tumor necrosis factor-α (TNF-α) and gastrointestinal hormones which include gastric inhibitory peptide (GIP), glucagon-like peptide-1 (GLP-1), somatostatin, serotonin (5-HIT), motilin (MTL) and gastrin. Blood samples stored by −80 °C were measured by Elisa assay (the vendor of kits is Jiangsu Yutong Biological Technology Co., Ltd.).

Data regarding complications, pathological outcomes, hospitalization cost and length of stay (LOS) were collected at discharge time.

### Sample size, randomization and statistics

Sample size calculation was performed for the main outcome, the time to first bowel movement, using the PASS 11.0 software. Randomization was performed using PASS 11.0 software to generate random numbers and divide patients into two group evenly, using opaque sealed sequentially numbered envelopes that were opened at the moment of surgery completion. Previous our studies on postoperative ileus after gastrectomy showed a mean time to the first bowel movement of 110 ± 25.7 h (mean ± standard deviation) for control patients [8]. To detect a clinically relevant absolute difference of 24 h in time to the first bowel movement with a two-sided significance level of 0·05 and a power of 90%, it was calculated that 26 patients per study arm would be required. Assuming a 20% dropout rate (withdrawal rate), 60 patients were required for the study. No interim analysis was planned or performed, and no early-stopping rules were implemented.

Continuous data are presented as the mean (s.d.). Time to first bowel sound, time to first flatus, time to first bowel movement and time to tolerance of solid food were assessed by *t*-test or ANOVA, as appropriate. Other continuous data were compared using the Mann–Whitney U test and categorical data with the *χ*^2^ test. *P* values were estimated with the likelihood ratio test. Two-sided *P* < 0.050 was considered statistically significant. Statistical analysis was performed using SPSS version 20.0 software (SPSS, Chicago, IL, USA).

### Ethics approval and trial registration

Ethical approval was given by the local ethics regional board: The Ethics Committee of Affiliated Hospital of Qingdao University. All patients involved in the trial signed informed consent. Registration with an approved clinical trials registry, the Chinese Clinical Trial Registry, was undertaken (ChiCTR1800018294).

## Results

**Fig. 1:**
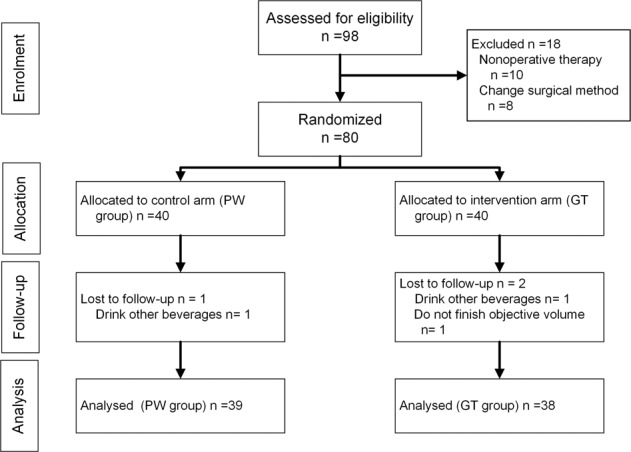
CONSORT flow diagram. 98 patients were screened consecutively for trial inclusion. After exclusion, a total of 80 patients were enrolled, gave consent and were allocated, and finally 77 patients were available for analysis (GT group *n* = 38, PW group *n* = 39).

Between September 2017 and September 2018, 98 patients were screened consecutively for trial inclusion. Of eligible patients, 10 patients received neoadjuvant chemotherapy on the decision by a multidisciplinary team, resulting in 88 patients receiving surgery therapy. After surgery, eight patients had a change of operation method: two patients received simple gastrojejunostomy for an unresectable tumor; three patients received hyperthermic peritoneal perfusion for intraperitoneal implantation and metastasis confirmed by intraoperative frozen sections; two patients received gastrectomy combined with partial transverse colectomy for transverse colon mesentery invasion; and one patient received total gastrectomy. A total of 80 patients were enrolled, gave consent and were allocated. Two patients (one in the GT group and one in the PW group) drank other beverages during the trial, and one patient in the GT group did not finish the minimum volume, resulting in 77 patients being available for the final analysis (see Fig. 1).

### Patient demographics and pathological data

Both patient groups were equally matched at baseline with regard to demographic data, with no significant difference in age, sex, BMI, ASA grade, NSR2002, comorbidities, smoking history, or indication for surgery. Pathological data show there was a difference in the type of signet-ring cell cancer, but the pathological stage of each of these five patients was T_1a_N_0_M_0_. We think this difference did not affect the results of our trial (see Table 2).

**Table 2:** Demographic, operative characteristics, and perioperative outcomes data.

Characteristic	GT group (*n* = 38)	PW group (*n* = 39)	*P*
Age —mean(range),y	58.42 ± 7.26	58.97 ± 9.91	0.901
Sex ratio — M:F	26:12	29:10	0.564^c^
BMI	25.72 ± 3.17	25.14 ± 4.62	0.522
Comorbidities—Y/N	11/27	14/25	0.515^c^
Smoker—Y/N	17:21	22:17	0.306^c^
ASA grade—(1/2/3)	7/29/2	4/34/1	0.533^a^
NRS2002 score—(1/2/3)	10/23/5	7/29/3	0.767^a^
Pathological type			
WDAC	0	1	1.0^b^
MDAC	7	13	0.136^c^
LDAC	26	25	0.689^c^
Signet-ring cell cancer	5	0	0.025^b^
Stage—(I/II/III)	19/6/13	18/10/11	0.969^a^
Number of lymph nodes	37.26 ± 14.23	32.21 ± 10.18	0.076
Vascular cancer embolus	5	12	0.098^c^
Perineural invasion	6	14	0.068^c^
Operation time (mean hours)	3.59 ± 0.83	3.90 ± 1.01	0.150
Blood loss (ml)	44.74 ± 20.89	52.31 ± 32.00	0.224
Intraoperative liquid volume	1713.16 ± 414.06	1710.53 ± 481.85	0.980
Laparoscopic/Robotic	19:19	21:18	0.736^c^
Anesthetic drug			
Propofol (g)	1.190 ± 0.31	1.241 ± 0.34	0.494
Fentanyl (mg)	84.08 ± 28.83	85.90 ± 27.05	0.776
Cisatracurium (mg)	21.16 ± 6.50	23.36 ± 9.20	0.230
vecuronium (mg)	10.32 ± 7.60	10.79 ± 8.76	0.799
Complication			
Complication rate	6 (15.79%)	11 (28.21%)	0.189
Pneumonia	2	5	0.249
Delayed gastric	1	3	0.615
Incision infection	0	2	0.484
Intestinal obstruction	1	0	0.494
Intraluminal bleeding	2	2	1.000
Venous tarombokinesis	0	1	1.000
30-d readmission	1	1	1.000
30-d mortality	0	0	1.000
Complication grades^d^			0.094^a^
1	8	12	
2	6	10	
3	0	0	
Symptoms			
Nausea	7	10	0.445
Vomiting	1	7	0.056^b^
Diarrhea	0	4	0.115^b^
Bloating	1	2	1.0^b^
Total	9	15	0.162
LOS in days	6.29 ± 0.927	7.05 ± 1.10	0.002

^a^Mann–Whitney U test.

^b^Fisher exact test.

^c^Chi-square test.

^d^Clavien-Dindo classification.

### Intraoperative parameters

There was no difference between the two groups with regard to operative data, as shown in Table 2.

### Postoperative complications

There was no difference in complication rate between the two groups. There were two cases of intraluminal bleeding in each group, which recovered after conservative treatment. There were three delayed gastric emptying in the cohort, one in the GT group and two in the PW group, with readmission within 30 days. There was no postoperative anastomotic leak in the sample. All patients in the GT group had no complications related to the intervention (see Table 2).

### Gastrointestinal function outcomes

**Fig. 2:**
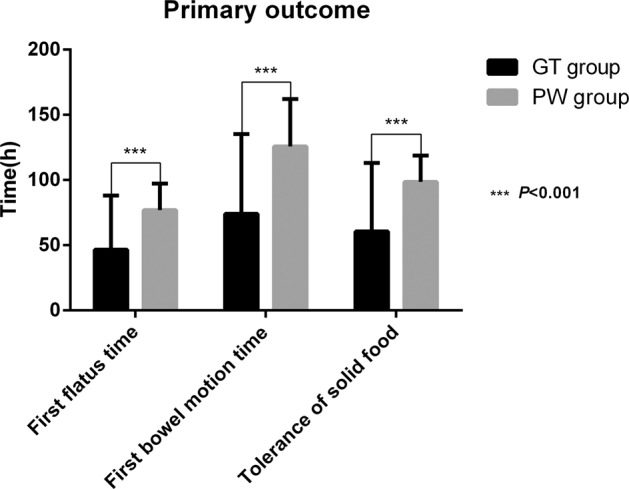
Time to first flatus, time to first bowel motion and time to tolerance of solid food between two group. Date are in mean as bar and whiskers as SD. The time to first flatus between the GT and PW groups was 47.23 ± 13.46 vs. 76.96 ± 20.35 (*P* < 0.001). The time to first bowel motion between the GT and PW groups was 78.70 ± 25.77 vs. 125.76 ± 36.25 (*P* < 0.001). The time to tolerance of solid food between the GT and PW groups was 62.20 ± 16.15 vs. 98.66 ± 20.15 (*P* < 0.001). Statistical method Mann–Whitney U test.

There was a significant difference in mean (mean hours ± SD) time to first flatus between the GT and PW groups (47.23 ± 13.46 vs. 76.96 ± 20.35, *t* = −7.580, 95% confidence interval(CI): −37.557~−21.897, *P* < 0.001). There was a significant difference in mean (mean hours ± SD) time to first bowel motion between the GT and PW groups (78.70 ± 25.77 vs. 125.76 ± 36.25, *t* = −6.557, 95%CI: −61.365~−32.766, *P* < 0.001). There was significant difference in mean (mean hours ± SD) time to tolerance of solid food between the GT and PW groups (62.20 ± 16.15 vs. 98.66 ± 20.15, *t* = −8.747, 95% CI: −44.761~−28.155, *P* < 0.001) (see Fig. 2).

### Length of stay and symptoms of nausea, vomiting, diarrhea and bloating

Postoperative hospital stay was significantly different between the GT group and PW group (mean days ± SD, 6.29 ± 0.93 vs. 7.05 ± 1.01, *t* = −3.283, 95% CI: −1.224~−0.300, *P* < 0.002). There was no difference in symptoms of nausea, vomiting, diarrhea or bloating (see Table 2).

### Analgesic outcome

**Fig. 3:**
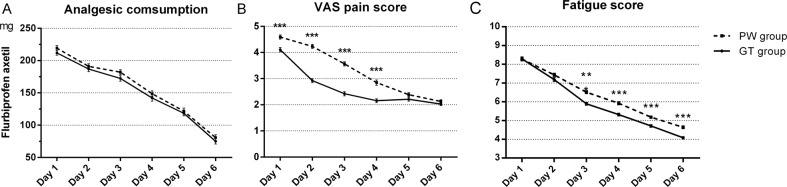
Flurbiprofen axetil consumption, VAS pain score and Fatigue scores between two group. Flurbiprofen axetil consumption (**A**) show there was no significance difference between two group and from day 1 to day 6 (*P* = 0.067). VAS pain score (**B**) show there was significance difference between two group and from day 1 to day 6 (*P* < 0.001). Fatigue scores (**C**) show there was significance difference between two group and from day 1 to day 6 (*P* < 0.001). Date are in mean as lines and whiskers as SEM, *t*-test *P* value, **P < 0.01, ***P < 0.001, Statistical method Repeated measures ANOVA.

Patients in the GT group had less perception of pain during postoperative day 1–day 4, and both groups had similar analgesic consumption rates in this study (see Fig. 3).

### Postoperative fatigue outcomes

Patients who drank GT had a lower degree of fatigue than the PW group, with significantly lower fatigue scores on days 3–6 (see Fig. 3).

### Gastrointestinal hormones

There was no difference in 5-HIT or gastrin between the two groups. GIP and somatostatin in the GT group was significantly lower than the PW group on day 3 and day 5 after surgery. Motilin and GLP-1 in the GT group were also significantly higher than the PW group on day 3 and day 5 after surgery (see Table 3).

**Table 3:** Biochemical indicators and inflammatory markers.

	GT group				PW group				*P*^a^
	Preop	Day 1	Day 3	Day 5	Preop	Day 1	Day 3	Day 5	
Indicators									
Total bilirubin (umol/L)	12.20 ± 4.73	15.47 ± 6.79	13.76 ± 5.51	11.43 ± 4.43	12.91 ± 7.31	15.71 ± 7.92	14.77 ± 6.88	10.65 ± 6.35	0.787
Albumin (g/L)	42.93 ± 3.15	34.39 ± 2.88	33.05 ± 3.11	32.50 ± 3.14	42.88 ± 5.56	34.58 ± 3.55	32.96 ± 3.32	32.53 ± 3.21	0.696
Prealbumin (mg/L)	363.20 ± 57.82	223.04 ± 47.59	154.06 ± 43.36	152.93 ± 49.10	279.05 ± 59.64	210.71 ± 53.67	146.61 ± 43.86	153.65 ± 45.45	0.447
Cholesterin (mmol/L)	4.94 ± 0.90	4.10 ± 0.72	4.26 ± 2.05	4.08 ± 1.11	5.11 ± 1.25	4.12 ± 0.84	3.91 ± 0.95	3.96 ± 0.84	0.710
Triglyceride (mmol/L)	1.35 ± 1.08	1.14 ± 0.64	1.17 ± 0.74	1.03 ± 0.50	1.52 ± 1.28	1.11 ± 0.62	1.27 ± 0.67	1.17 ± 0.39	0.504
HDL (mmol/L)	1.35 ± 0.32	1.18 ± 0.27	1.22 ± 0.27	1.03 ± 0.22	1.42 ± 0.32	1.26 ± 0.26	1.32 ± 0.51	1.08 ± 0.24	0.183
LDL (mmol/L)	2.99 ± 0.77	2.42 ± 0.62	2.05 ± 0.51	2.38 ± 0.66	3.16 ± 0.99	2.42 ± 0.68	2.01 ± 0.65	2.28 ± 0.82	0.949
LP(a) (mg/L)	265.16 ± 189.30	247.12 ± 171.72	257.59 ± 161.90	307.42 ± 195.14	247.26 ± 139.40	261.34 ± 171.74	273.03 ± 150.10	318.69 ± 190.65	0.897
Fasting blood-glucose (mg/dL)	94.50 ± 25.56	113.76 ± 23.76	100.26 ± 22.14	101.70 ± 20.16	93.78 ± 23.22	130.14 ± 48.24	110.16 ± 25.56	103.86 ± 27.90	0.117
Mg (mmol/L)	0.95 ± 0.05	0.90 ± 0.08	0.88 ± 0.09	0.87 ± 0.09	0.95 ± 0.05	0.91 ± 0.07	0.87 ± 0.07	0.86 ± 0.06	0.662
Ca (mmol/L)	2.23 ± 0.14	2.04 ± 0.07	2.05 ± 0.08	2.06 ± 0.10	2.24 ± 0.13	2.03 ± 0.08	2.03 ± 0.06	2.04 ± 0.07	0.403
Na (mmol/L)	141.13 ± 1.94	138.95 ± 2.08	139.53 ± 2.18	140.50 ± 2.14	141.21 ± 3.05	139.33 ± 2.82	139.08 ± 2.74	140.38 ± 3.08	0.381
K (mmol/L)	4.30 ± 0.33	4.38 ± 0.31	4.19 ± 0.31	4.05 ± 0.39	4.27 ± 0.33	4.40 ± 0.40	4.14 ± 0.33	3.99 ± 0.52	0.629
Markers									
Leucocyte (10^9^/L)	6.00 ± 1.62	11.00 ± 2.38	8.37 ± 2.90	6.78 ± 2.13	6.52 ± 1.42	11.64 ± 3.14	8.06 ± 2.93	7.13 ± 1.72	0.432
Neutrophils percent	54.31 ± 9.91	83.13 ± 5.28	76.22 ± 8.85	69.38 ± 7.83	55.31 ± 8.86	83.11 ± 6.15	74.31 ± 9.57	69.31 ± 7.41	0.842
CRP (mg/L)	1.93 ± 1.25	14.58 ± 9.90	69.58 ± 38.12	37.74 ± 27.31	3.36 ± 5.90	19.82 ± 15.15	66.38 ± 35.98	33.18 ± 19.24	0.948
PCT (ng/ml)	0.021 ± 0.003	0.109 ± 0.087	0.161 ± 0.138^*^	0.116 ± 0.147	0.021 ± 0.004	0.175 ± 0.229	0.279 ± 0.334	0.160 ± 0.157	0.011
IL-6 (pg/ml)	24.16 ± 4.22	40.34 ± 4.03	53.52 ± 4.64	34.55 ± 3.08	25.10 ± 3.87	41.62 ± 4.14	53.79 ± 4.45	34.56 ± 3.88	0.206
TNF-α (pg/ml)	41.11 ± 8.78	56.98 ± 9.01	51.58 ± 7.20^***^	38.00 ± 4.33^***^	43.08 ± 6.08	55.18 ± 8.84	62.13 ± 5.32	48.08 ± 4.22	<0.001
GastrointestiHormone									
GIP (pg/ml)	382.23 ± 61.72	344.81 ± 35.98	315.35 ± 20.50^***^	330.41 ± 35.32^**^	383.10 ± 60.72	335.20 ± 39.16	368.47 ± 38.09	356.97 ± 40.56	0.002
GLP-1 (pmol/ml)	7.00 ± 0.62	7.17 ± 0.52	10.17 ± 0.82^***^	9.45 ± 0.70^*^	7.12 ± 0.61	7.30 ± 0.61	8.61 ± 0.71	9.31 ± 0.92	<0.001
5-HIT (pg/ml)	1549.29 ± 179.08	1621.91 ± 255.28	1412.43 ± 217.99	1181.19 ± 124.19	1606.07 ± 132.26	1631.00 ± 271.62	1387.78 ± 229.07	1231.88 ± 129.87	0.44
MTL (pg/ml)	358.50 ± 31.16	304.10 ± 46.29	309.50 ± 32.66^***^	302.44 ± 29.97^***^	364.96 ± 37.23	320.20 ± 48.67	261.99 ± 49.42	271.77 ± 24.26	0.26
Gastin(pg/ml)	122.13 ± 22.77	175.37 ± 16.54	181.42 ± 13.42	179.26 ± 26.65	128.00 ± 24.53	180.05 ± 19.04	187.10 ± 13.08	186.90 ± 18.84	0.062
Somatostain (pg/ml)	27.36 ± 2.94	31.12 ± 2.74	30.38 ± 3.20^***^	30.77 ± 2.71^***^	26.71 ± 2.75	32.12 ± 3.71	35.84 ± 3.48	35.07 ± 3.24	<0.001

*t*-test *P* value.

*P < 0.05; **P < 0.01; ***P < 0.001.

^a^Repeated measures ANOVA *P* value.

### Biochemical indicators and inflammatory markers

There was no difference in biochemical indicators between the two groups. There was no difference in leukocyte count, percentage of neutrophils, CRP or IL-6. There was significant difference in PCT or TNF-α at postoperative day 3 (see Table 3).

### Adverse events

Compliant patients in the intervention group tolerated the GT after surgery, and there were no adverse events or complications related to drinking GT. No unplanned analyses were performed.

## Discussion

The results showed that drinking Chinese GT after surgery did not increase the LOS, and the incidence of anastomosis leakage or other postoperative complications compared with the control group. No patient died within 30 days of operation. There was no significant influence on liver function, fasting blood glucose, lipid metabolism or water-electrolyte balance. There was no difference in overall symptoms of nausea, vomiting, diarrhea or bloating between the two groups. Thus, GT consumption after gastrectomy was safe enough.

TNF-α reflects the active degree of systemic inflammation, and PCT is a parameter for diagnosing and monitoring bacterial inflammatory disease infection. The magnitude of the elevation correlates with the severity of infection. The lower serum PCT and TNF-α in GT group than PW group revealed that drinking Chinese GT after gastrectomy may possess a certain anti-infective effect and reduce the postoperative inflammatory reaction. This would be consistent with previous in vitro or animal experiments that tea catechins have anti-inflammatory and anti-bacterial activity [15], and these responses have been described in humans [16], but there are no reports of this effect after surgery, which suggest the need for further intensive study.

In our trial, patients were received flurbiprofen axetil 100 mg bid from postoperative day 1 to day 3, increasing or decreasing according to the pain tolerance of patient. The result revealed that VAS scores had a significantly different on days 1–4, when patients received flurbiprofen axetil treatment. Daily analgesic consumption was similar in the two groups, but the analgesic effect was better in the GT group than the PW group. NSAID and caffeine co-administration can improve the analgesia efficacy with a dose-effect relationship and concentration dependence [26, 27]. The reason for the analgesic superiority in the GT group may be the synergistic action of NSAIDs and caffeine.

Patients in the intervention group had a lesser degree of fatigue than the control group, with significantly lower fatigue scores on days 3–6. Green tea increases wakefulness on account of the anti-fatigue effects of caffeine and catechins. Caffeine can act specifically within the central nervous system to delay fatigue by blocking adenosine receptors, and catechins can alleviate the fatigue caused by exercise [28]. We required early mobilization after surgery and increasing amounts of exercise according to the degree of pain tolerance and physical strength, but the motivation for out-of-bed activity in patients was weakened by postoperative fatigue syndrome. Patients who drank GT had a quicker recovery from fatigue after surgery and good compliance with early mobilization, which enhanced postoperative recovery.

We observed that patients in the GT group had superior postoperative gastrointestinal functional recovery, with significantly shorter times to first flatus, first bowel motion and tolerance of solid food. This phenomenon may have several explanations. First, caffeine in GT can stimulate bowel motility [12]. Gastrointestinal smooth muscle responses depend upon excitatory and inhibitory neurotransmitters, and caffeine enhances vagal autonomic nerve activities [29], releasing acetylcholine, which is the most prominent excitatory neurotransmitter [30]. Secondly, neuronal NO is an important inhibitory regulator of gastrointestinal motility, and at the level of the stomach, NO is involved in non-adrenergic non-cholinergic relaxation of the fundus and pylorus. Inhibition of nitric oxide syhthase (NOS) delays gastric emptying of liquids in rats and solids in dogs [31]. And this effect of NO can be partially reversed by tea extract [32] and L-theanine a component of GT [33], which indicate GT could reverse the inhibited role of NO through decreasing restoration of the intestinal NOS activity. Besides, tea extract and thearubigins increased gut motility and this prokinetic effect was reversed by ondansetron (a 5-HT_3_ antagonist) indicating that tea extract and thearubigins could be considered as 5-HT_3_ receptors agonists [34]. Finally, neurohumoral regulation may play an important role in the process of recovery.

Moreover, we tested the gastrointestinal hormones and other factors on postoperative day and days 1, 3 and 5 after surgery. GIP reduces intestinal motility in mice [35], but in humans this effect is absent in normal conditions [36]. Although the PW group had a high level of GIP, it seems not to be the reason for the slow recovery of gastrointestinal function. After gastric bypass surgery, postprandial plasma GLP-1 can increase substantially [37], and the effects of GLP-1 on intestinal motility in human are weaker [38]. In our trial, patients received Roux-en-Y gastrojejunostomy, and the GT group had a short time to tolerance of solid food. These factors may have led the GT group to have high GLP-1 [39]. Somatostatin affects gastrointestinal motility, delaying the gastric emptying and colon activity [40]. The GT group had lower somatostatin than the PW group on day 3–day 5. Caffeine intake enhances vagal autonomic nerve activities [29], and acetylcholine from vagal fibers inhibits somatostatin release. Caffeine from GT may activate the vagal nerve, inhibits somatostatin secretion, leading to a fast recovery of gastrointestinal function. Motilin induces gastric-origin phase III MMC activity, and GI motility is closely parallel with the secretion of motilin [41, 42], and we found high plasma motilin in the GT group on day 3–day 5. However, neurohumoral regulation is a complex process, and the present study on neurohumoral regulation mechanisms of GT is very preliminary. More details need to be illuminated in the future.

Our analysis has certain limitations. Its nature as a single-center study and its small sample size may have limited the statistical power to detect differences in postoperative complications. In addition, the intervention has unique smell and taste, and it is difficult to be concealed, which may lead to social desirability bias. Besides, there are diverse factors influencing the gastrointestinal function around perioperative duration which may dilute the effect of GT on patients. Therefore, we kept other quantities coincidently in two group to control the confounding bias. Moreover, mechanisms of enhancing GI function recovery for GT are short of conclusive evidences. In order to reduce bias in the effects of basic diseases on GI function, we excluded patients with diabetes, thyroid disease, or previous extensive abdominal surgery. Thus, we were not able to describe the true conditions of patients in the clinic, but future research should aim to do so.

The concept of ERAS programs has had a major impact on surgical care all over the world, which lead to major reduction in postoperative hospitalization, medical complications and convalescence across surgical procedures. The results represent a unique combination of improved quality with concomitant reduction of costs. In the future, drinking GT after operation may become one of the measures to promote gastrointestinal function after abdominal surgery in ERAS management for the reasons that it is appropriate to clinic and easily generalized for availability, feasibility, acceptability and low-cost.

## Conclusion

In summary, this study reveals that drinking GT was a safe intervention and enhanced the recovery of patients after laparoscopic or robotic subtotal distal gastrectomy for gastric cancer, also was an add method with strengthening analgesia and anti-inflammatory effect in the presence of the ERAS program.

## Supplementary information

CONSORT 2010 Checklist
